# Transient Disturbance and Community Convergence of Coral-Associated Microbial Communities Under Acidification, Benzo[*a*]pyrene Pollution, and Combined Stress in Coral *Acropora millepora*

**DOI:** 10.3390/microorganisms14102259

**Published:** 2026-10-04

**Authors:** Zhao Qi, Yumei Chen, Lianzheng Yin, Hongmei Jing, Chuanliang Wu, Tinghan Yang, Xiaoping Diao

**Affiliations:** 1 College of Ecology, Hainan University, Haikou 570228, China; 2 State Key Laboratory of Marine Resources Utilization in South China Sea, Haikou 570228, China; 3 School of Life and Health Sciences, Hainan University, Haikou 570228, China; 4 State Key Laboratory of Deep-Sea Science and Intelligent Technology, Institute of Deep-Sea Science and Engineering, Chinese Academy of Sciences, Sanya 572000, China; 5 Sanya Institute of Coral Reef Ecology Research, Sanya 572000, China; 6 Guangxi Key Laboratory of Beibu Gulf Marine Resources, Environment and Sustainable Development, Fourth Institute of Oceanography, Ministry of Natural Resources, Beihai 536000, China

**Keywords:** coral-associated microbiomes, acidification, benzo[*a*]pyrene, Acid + BaP stress, core microbes

## Abstract

Ocean acidification (OA), driven by global climate change together with pollution from organic contaminants, is increasing the ecological risk faced by coral reef ecosystems. The coral holobiont comprises diverse microbial communities that collectively support the coral host in coping with and adapting to environmental stress. However, the response of the coral holobiont to the combined stress of acidification and benzo[*a*]pyrene (BaP) remains poorly understood. In this study, *Acropora millepora* was selected as a model species to investigate the responses of coral-associated microbiomes to acidification (pH 7.8), BaP exposure (10 µg·L^−1^) and their combined effects. We found that while the cell density of coral-associated Symbiodiniaceae increased significantly with prolonged exposure time in all treatment groups, and there were no temporal differences in chlorophyll-a contents. The Symbiodiniaceae community was stable within all treatments, with *Cladocopium* as the dominant genus, and subtype C3u had the highest relative abundance (>70%). A rapid and significant increase in rare bacteria taxa was observed in the single-stress treatments (acidification, BaP), whereas prolonged exposure led to an increasing number of unique bacteria taxa in the combined-stress group in two weeks. Surprisingly, the main community structure of core coral-associated bacteria in the combined treatment group was close to that of the control group. Across all treatments, core coral-associated bacteria (including Sphingomonadaceae and Burkholderiaceae) consistently dominated the microbial community, while core coral-associated archaea exhibited more stochastic variations. Collectively, these results highlight the critical role of coral-associated microbiomes in rapidly and flexibly responding to environmental stressors, thereby contributing to the maintenance of holobiont stability under multiple stress conditions.

## 1. Introduction

Coral reef ecosystems are among the most productive ecosystems on Earth. Owing to their exceptional ecological and socio-economic value, they have attracted extensive global attention [1,2]. However, coral reef ecosystems are facing the impacts of global climate change and human activities. In recent years, large-scale coral bleaching events have been reported worldwide [3]. While considerable efforts have been devoted to coral conservation and restoration, it is equally critical to understand how coral holobionts respond to the complex and rapidly changing environmental conditions. The coral holobiont represents a highly integrated biological unit composed of the coral host and its associated microorganisms, including bacteria, archaea, Symbiodiniaceae, and viruses, all of which are essential to coral health and resilience [4,5]. Coral-associated microorganisms supplement key host functions, such as the nitrogen cycle and sulfur cycle [6], and coral health is therefore closely linked to the structure and stability of its microbial communities. Under the combined pressures of excessive atmospheric CO_2_ emissions and anthropogenic activities, ocean acidification [7] and organic pollutant contamination [8] have emerged as two major environmental stresses affecting coral reefs. It has been projected that the average pH of surface seawater may decline to approximately 7.8 by 2100 [9,10]. Concurrently, environmental pollution by polycyclic aromatic hydrocarbons (PAHs), characterized by low bioavailability and resistance to degradation, has increased with industrialization and urbanization [11]. Benzo[*a*]pyrene (BaP), a representative high-molecular-weight PAH widely distributed in coral reef ecosystems [12], is well known for its carcinogenic, teratogenic and toxic properties. Derived primarily from fossil fuel combustion and vehicle exhaust emissions, BaP can accumulate in environmental media and biological tissues, thereby including toxicity and contributing to coral bleaching. While other pollutants (e.g., metals and herbicides) may also be present at this site, BaP was selected as a representative PAH due to its widespread occurrence in the study area and its known toxicity to reef organisms.

Declining seawater pH has been shown to significantly affect coral physiology and symbiosis. Experimental studies have demonstrated that under pH 7.6 conditions, *Acropora* sp. experience 40–50% bleaching [13]. Similarly, Ge et al. reported that under ocean acidification, particularly at pH 7.8 or lower, the cell density of coral-associated Symbiodiniaceae declines significantly [14]. Collectively, previous studies suggest that pH 7.8 represents a critical threshold for maintaining microbial stability in *Acropora* sp. [15,16]. Coral-associated microorganisms actively participate in coral responses to acidification, influencing host health through dynamic shifts in community composition. Although Symbiodiniaceae and other microbial taxa can cooperate with the host to mitigate acidification stress, coral responses vary considerably among species, and the underlying resistance mechanisms differ. For example, Symbiodiniaceae cell density in tropical and subtropical corals has been shown to be negatively correlated with the degree of ocean acidification [17]. Acidification can also promote the proliferation of pathogenic bacteria while reducing beneficial microbial taxa, leading to pronounced changes in the coral-associated bacterial community [15]. Nevertheless, some coral–microbe associations and physiological parameters remain relatively stable under acidified conditions [18], highlighting the complexity of coral microbial responses and the need for further investigation.

Persistent organic pollutants, particularly polycyclic aromatic hydrocarbons (PAHs), represent another long-standing threat to marine ecosystems. These compounds can enter the ocean through atmospheric deposition, runoff, and groundwater discharge [19], ultimately accumulating in marine organisms. Benzo[*a*]pyrene (BaP), as a typical PAH, has attracted particular concern due to its widespread presence in coral reef regions and its strong lipophilicity, which facilitates bioaccumulation [12]. Upon entering coral tissues, BaP can trigger detoxification responses, disrupt metabolic processes, and impair the photosynthetic efficiency and cell density of Symbiodiniaceae in *Acropora* sp. corals, ultimately destabilizing the coral–algal symbiosis. Previous monitoring studies have reported elevated PAH concentrations in coastal waters in China [20,21]. The seawater collected from Hainan for this experiment contained total PAHs at concentrations ranging from 0.28 to 0.36 μg·L^−1^, while our nominal exposure concentration was 10 μg·L^−1^, approximately 20–40 times higher than reported environmental levels.

In natural marine environments, ocean acidification and organic pollutant contamination frequently occur simultaneously. Acid–base conditions can influence the toxicity, bioavailability, and environmental behavior of organic pollutants, while acidification itself may alter their biological effects [22]. Therefore, studies focusing on combined stressors provide a more realistic assessment of coral responses than single-factor experiments. However, there are few researchers who have reported the acidification–BaP combined stress on corals. Our previous study confirmed that BaP is widely present in the seawater, sediments and corals in Hainan [20,21], where it constitutes a major component of high-ring PAHs [21]. Notably, exposure to BaP at 10 µg·L^−1^ induced significant changes in gene expression in *Acropora* sp. corals. In the present study, combined acidification–BaP stress was employed to simulate complex reef environmental conditions and to investigate the responses of coral-associated microbial communities. Acidification was set at pH 7.80 and BaP concentration at 10 µg·L^−1^, with the aim of elucidating microbial community dynamics and adaptive patterns under prolonged exposure. This study provides insights into the dynamic balance between coral-associated microbial community structure and environmental change and offers theoretical support for understanding the role of microbial assemblages in maintaining coral homeostasis under multifactorial stress.

## 2. Materials and Methods

### 2.1. Study Site and Materials

*Acropora millepora*, one of the most abundant reef-building coral species in Hainan, China, was selected as the study organism. Coral colonies were collected in June 2021 from the reef area of Phoenix Island, Sanya, China (109°29′8.06″ S, 18°14′26.64″ N). Corals were immediately brought back to the laboratory after collection, decomposed into broken fragments of about 3–5 cm, and continued for one week of adaptive domestication. The stress experiment was conducted in tanks containing 5 L of seawater. Corals were farmed using natural seawater that had been settled and filtered through the 0.45 µm filter membrane, the coral housing room temperature was 25 ± 0.5 °C, the salinity was 34 to 35%, the pH was 8.10–8.30, with a fixed 12 h of light and 12 h of darkness every day, and the photoperiod was provided using LED aquarium lamps (25 W, 300 μmol photons·m^−2^·s^−1^ photosynthetically active radiation).

### 2.2. Experimental Design

The BaP stock solution was prepared with benzo[*a*]pyrene (BaP) (purity ≥ 96%, Sigma-Aldrich, St. Louis, MO, USA) and DMSO (purity ≥ 99.5%, Xilong Science, Guangdong, China), and was ready for use in the exposure experiment. Based on the previous research results of our laboratory and relevant literature reports [12,23] and the concentration distribution of PAHs in the coral reef area of Hainan [21], the experimental groups were the seawater control group (control), DMSO solvent control group (DMSO), pH 7.80 acidification treatment group (Acid), 10 µg·L^−1^ BaP treatment group (BaP), and acidification–BAP combined treatment group (Acid + BaP). An equal volume of DMSO (purity ≥ 99.5%) was added to the solvent control group, corresponding to the amount used in the BaP treatment group. All experimental treatment groups were set up with 3 parallel tanks, and 9 to 16 coral fragments were placed in each group. Each fragment was about 3–5 cm in length and was secured to the base tray, placed evenly on the plastic mesh plate at the bottom of the tank. There were at least 3 colonies in each tank.

During the experiment, the seawater temperature, salinity and light time remained consistent with those during the adaptive domestication period. Referring to the results of the pre-experiment and the previous stress research results in the laboratory [12,24], as well as the literature reports [23], it was determined to collect the coral samples on the seventh and fourteenth days of the exposure experiment, then immediately transfer them to a −40 °C refrigerator for storage, for the subsequent extraction of DNA and measurement of physiological indicators. For each group, the physiological indicators and DNA extraction of the 3 biological replicates (n = 3, taken one fragment from each parental tank) were analyzed at each time point.

Acidification stress pH control: CO_2_ was injected using a CO_2_ enrichment device (State Key Laboratory of Marine Environment, Xiamen University, CE100D model, Wuhan Ruihua Instrument & Equipment Co., Ltd., Hubei, China) with a concentration set at 1108 ppm, and a pH meter (Shanghai Mettler-Toledo Instrument Co., Ltd., Shanghai, China) was used for monitoring. BaP stress concentration control: The final concentration of the solvent in the BaP treatment group should be less than oneten-thousandth of the volume of seawater to avoid solvent interference. All the seawater was replaced at regular intervals every day, and a fixed volume of BaP liquid was added to the BaP stress group and the combined-stress group respectively to ensure that the BaP concentration was consistent every day. During the exposure, pH was maintained (±0.01 unit) using a CO_2_ bubbling system with calibrated monitoring.

### 2.3. Microbial Properties

Total DNA extraction from microorganisms: We weighed approximately 0.5 g of each coral sample, ground it into powder with liquid nitrogen, placed the powder in a 2 mL centrifuge tube, and added the pre-prepared and sterilized DNA lysis buffer. This was shaken thoroughly (about 5 min) with 10 mM Tri-HCl pH 8.0, 100 mM EDTA pH 8.0, 0.5% *w*/*v* SDS until coral tissue and algae were separated from the samples. Then, we placed the sample solution in a 56 °C metal bath for 1–1.5 h for enzymatic hydrolysis incubation. Efficient grinding was carried out using a high-throughput tissue grinder. After re-incubation to ensure that the cells were basically completely ruptured, total DNA was extracted using the CTAB method and purified using the Zymo DNA Clean & Concentrator kit (Zymo Research, Irvine, CA, USA), and then the concentration and quality were determined using a NanoDrop ultramicent ultraviolet spectrophotometer (Thermo Fisher Scientific, Waltham, MA, USA) and finally stored at −20 °C.

### 2.4. Physiology and Biochemistry Characterization

The surface of the coral skeletons was repeatedly rinsed with artificially prepared seawater in small amounts until they were completely bleached. The bleached coral skeletons were kept for future use. We passed the rinsed seawater through a 300-mesh sieve and collected the filtrate into a 50 mL centrifuge tube. We pre-cooled the centrifuge to 4 °C in advance, balanced the filtrate centrifuge tubes and placed them in the pre-cooled centrifuge. We set the centrifugation conditions to 4000 g and centrifuged for 10 min. After centrifugation, the supernatant was removed. The cell suspension was made up to 10 mL with artificial seawater and mixed well for later use. This cell suspension was used for the determination of cell density of zooxanthella and chlorophyll-a [21]. The determination of cell density using disposable blood count frame count took 10 μL of cell suspension in order to count the cells with a biological microscope (Motic, Xiamen, China), and detection, according to the concentration of zooxanthella calculation formula to calculate the volume per ml containing the cell number, used the total cell number divided by the coral bone surface area; we finally obtained the cell density per unit coral skeletal surface area (cell·cm^−2^). Coral fragment surface area was estimated using the aluminum foil wrapping method. Zooxanthella chlorophyll content determination was carried out using acetone extraction, which was then joined with the determination of liquid, such as 90% of the volume of acetone, avoiding light, which was stored in −20 °C for 24 h and then centrifuged to remove impurities. Spectrophotometer absorbance was used to calculate the determination, and the formula for computing [25] chlorophyll-a content is shown below. The absorbance of the supernatant was measured at 663 nm and 645 nm, and chlorophyll-a content was homogenized using the surface area of coral skeletons; the unit was μg·cm^−2^.

Chlorophyll-a = 12.7 A663 − 2.69 A645

### 2.5. Sequencing of High-Throughput Libraries

High-throughput sequencing generated an average around of 50,000 reads per sample. PCR amplification of DNA was performed using the Sym-ITS2.F (5′-GTGAATTGCAGAACTCCGTG-3′) and Sym-ITS2.R (5′-CCTCCGCTTACTTATATGCTT-3′) primers specific for Symbiodiniaceae [26,27]. Library preparation was outsourced to Novogene Bioinformatics Technology Co., Ltd. (Beijing, China) using the TruSeq^®^ DNA PCR-Free Sample Preparation Kit (Illumina, Inc., San Diego, California, USA). High-throughput sequencing was performed on the NovaSeq PE250 platform (Novogene Co., Ltd., Beijing, China). The PCR amplification system (20 μL) was as follows: 10 μL 5×PrimeSTAR Buffer (Mg^2+^ plus), 4 μL dNTP Mixture (2.5 mM), 2 μL primer (0.1 μM), 0.5 μL PrimerSTAR HS DNA Polymerase, 10 ng DNA as a template, and ddH_2_O, added finally to bring the total reaction volume to 20 μL. The PCR reaction program consists of a 5 min pre-denaturation step at 94 °C, followed by 30 cycles (denaturation at 95 °C for 30 s, annealing at 52 °C for 30 s, and extension at 72 °C for 1 min), concluding with a final extension at 72 °C for 10 min.

The 16S sequencing and library preparation of coral-associated bacteria and archaea were performed by Novogene Bioinformatics Technology Co., Ltd. (Beijing, China). The V3–V4 region of the 16S ribosomal DNA gene was amplified using bacterial primers 341F (5′-CCTAYGGGRBGCASCAG-3′) and 806R (5′-GGACTACNNGGGTATCTAAT-3′) [28]. The V4 region of the 16S rRNA gene was amplified using the archaeal primers Arch519F (5′-CAGCCGCCGCGGTAA-3′) and Arch915R (5′-GTGCTCCCCCGCCAATTCCT-3′) [29]. We amplified the gene fragment from each sample using three 25 μL PCR reactions. The reaction system included forward and reverse primers (0.1 μM), 2× Taq PCR Mix buffer (Takara, Japan), and 10 ng total DNA. PCR Protocol: (1) 95 °C for 5 min; (2) 34 cycles, each cycle comprising 94 °C for 1 min, 55 °C for 1 min, and 72 °C extension for 1 min; and (3) 72 °C for 10 min, followed by 16 °C for 5 min. Samples were then transported on dry ice to Novogene Bioinformatics Technology Co., Ltd. in Beijing for sequencing analysis.

### 2.6. High-Throughput Sequencing Data Processing

Raw sequencing data from Symbiodiniaceae were processed by removing barcodes and primers. Quality control of ITS2 raw reads obtained from the platform was performed using Mothur software (version 1.34.4). Assembly and filtering yielded high-quality tag data. The UCHIME tool within Mothur was then applied to remove chimeras, yielding effective tags. Community composition at the genus and species levels were determined through ITS2 sequence alignment analysis.

Microbial high-throughput data analysis utilized the Illumina NovaSeq PE250 sequencing platform (Novogene Co., Ltd., Beijing, China). Quality control procedures followed QIIME (V1.9.1) protocols [30], employing tag trimming, length filtering, and chimera removal to ensure sequence integrity. All effective tags across all samples were clustered. By default, sequences were grouped into OTUs (Operational Taxonomic Units) at 97% sequence identity. Following the algorithm’s principles, the most frequently occurring sequence within each OTU was selected as its representative sequence. Species annotation was performed on OTU sequences using the Mothur method and the SILVA 138 SSU rRNA database [31]. Single sequences and non-target taxa were subsequently removed.

### 2.7. Statistical Analyses

Data are expressed as mean ± standard deviation. Normality of variances was assessed for the physiological data (or the standardized residuals of the model) using Shapiro–Wilk tests complemented with visual examination of Quantile–Quantile Plots, and homogeneity of variances was assessed using Levene’s test. The results indicated approximate normality and equal variances across all comparisons (*p* > 0.05 for all). One-way ANOVA followed by Tukey‘s HSD post hoc test was used as the primary approach to compare physiological indicators among groups. The Kruskal–Wallis H test was additionally performed as a robustness check, with Dunn’s test for pairwise comparisons and Bonferroni correction applied for multiple comparisons. To evaluate the effects of treatment and time on the indicators, a two-way ANOVA was performed, followed by Tukey‘s HSD post hoc test for multiple comparisons.

All analyses were performed in IBM SPSS Statistics 26.0 (IBM Corp., Armonk, NY, USA). Analysis of beta diversity analysis was conducted based on the Bray–Curtis distance matrix. OTU counts were converted to relative abundances (each sample summing to 1) prior to beta diversity analysis to account for sequencing depth variation. Bray–Curtis distances were calculated on the relative abundance data. To visualize the clustering patterns of samples, Principal Coordinate Analysis (PCoA) was performed for each time point separately. Permutational Multivariate Analysis of Variance (PERMANOVA) was conducted using the adonis2 function (R package vegan, version 2.6-6.1) with 9999 permutations. Analysis of Similarities (ANOSIM) was simultaneously applied using the anosim function, which tests the hypothesis that the rank of between-group dissimilarities is greater than that of within-group dissimilarities. To further investigate the influence of both experimental time and treatment, as well as their interaction effect on the microbial community, we employed a multi-factor PERMANOVA to test the main effects of time and treatment. Pairwise comparisons were carried out using permutation MANOVAs on a distance matrix with 9999 permutations. For microbial relative abundance, one-way ANOVA with Dunnett’s post hoc test was used when normality and homogeneity of variance were satisfied; otherwise, the Kruskal–Wallis test with Dunn’s post hoc test and Bonferroni correction was applied. Sequencing data and analytical results were visualized using RStudio 2024.04.2+764 and Excel^®^ 2021.

## 3. Results

### 3.1. Response of Coral-Associated Symbiodiniaceae Density and Chlorophyll-a Content to Different Stresses

**Figure 1:**
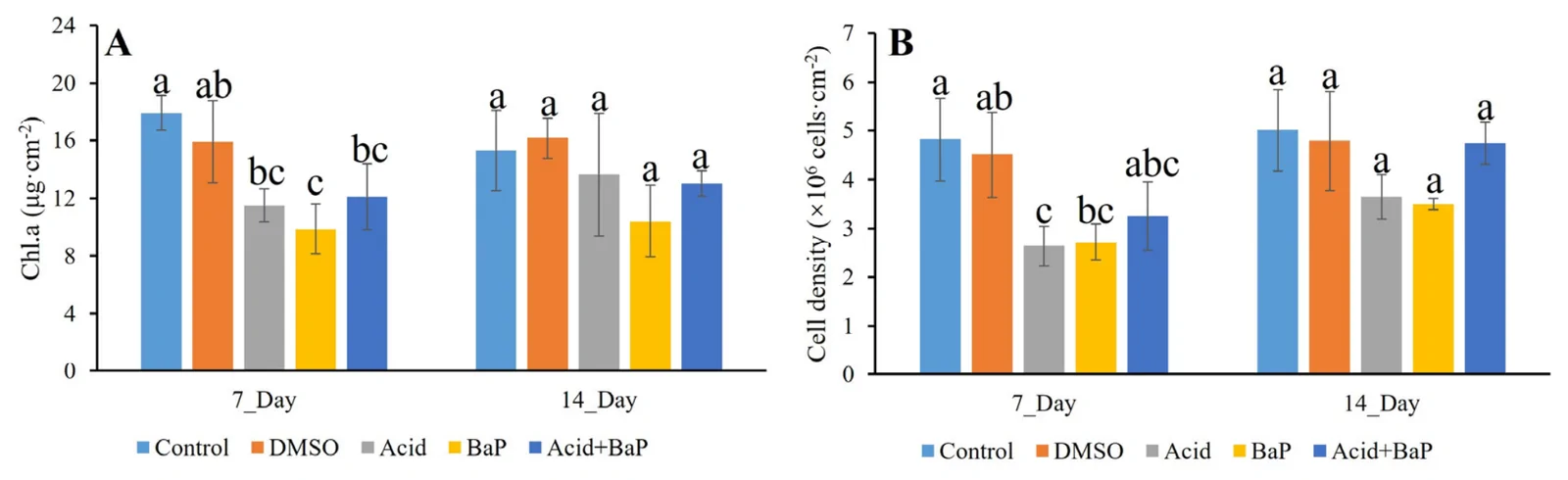
Changes in Chl.a (**A**) and cell density (**B**) of coral-associated Symbiodiniaceae under different stresses. Different superscript letters (a, b, c) indicate significant differences between groups (*p* < 0.05, Tukey’s HSD post hoc test).

Chlorophyll-a (chl-a) content of coral-associated Symbiodiniaceae differed among treatment groups (Figure 1A). On day 7 of exposure, Levene’s test confirmed homogeneity of variances (*F*(4, 10)= 1.415, *p* = 0.298), and chl-a contents revealed a significant difference among groups (one-way ANOVA, *F*(4, 10)= 8.671, *p* = 0.003; Kruskal–Wallis, H = 10.600, df = 4, *p* = 0.031). The chl-a contents in the control and DMSO control groups were 17.93 ± 1.23 µg·cm^−2^ and 15.93 ± 2.86 µg·cm^−2^ respectively. Post hoc Tukey HSD test showed that significantly lower chl-a contents were observed in the acidification (11.53 ± 1.15 µg·cm^−2^), BaP (9.89 ± 1.75 µg·cm^−2^) and Acid + BaP (12.14 ± 2.27 µg·cm^−2^) treatments (*p* < 0.05) compared with the control group. Notably, chl-a content in the combined treatment was higher than in either single-stress treatment. By day 14, Levene’s test confirmed homogeneity of variances (*F*(4, 10)= 2.491, *p* = 0.110), and there was no significant difference among groups in chl-a content (one-way ANOVA, *F*(4, 10)= 2.136, *p* = 0.151; Kruskal–Wallis, H = 7.267, df = 4, *p* = 0.122). The contents of control and DMSO control groups were 15.32 ± 2.79 and 16.17 ± 1.41 µg·cm^−2^ respectively. The BaP treatment showed the lowest value of chl-a content (10.43 ± 2.49 µg·cm^−2^), while slight decreases were observed in the acidification and Acid + BaP treatments compared with the control group.

Levene’s test confirmed homogeneity of variances (*F*(4, 10)= 1.240, *p* = 0.355), and cell density of coral-associated Symbiodiniaceae varied significantly (one-way ANOVA, *F*(4, 10) = 6.777, *p* = 0.007; Kruskal–Wallis, H = 9.833, df = 4, *p* = 0.043) among treatments on day 7 and exhibited a clear time-dependent pattern (Figure 1B). On day 7, cell density in the control and DMSO control groups were (4.8 ± 0.85) × 10^6^ cells·cm^−2^ and (4.51 ± 0.88) × 10^6^ cells·cm^−2^ respectively. In comparison with the control group, significantly lower densities were observed in the acidification ((2.64 ± 0.41) × 10^6^ cells·cm^−2^) and BaP ((2.72 ± 0.38) × 10^6^ cells·cm^−2^) treatments (*p* < 0.05), whereas the Acid + BaP group ((3.25 ± 0.71) × 10^6^ cells·cm^−2^) did not differ significantly from the control or DMSO control groups, consistent with the chl-a trends. On day 14 of exposure, Symbiodiniaceae cell density increased across all treatments, reaching (5.01 ± 0.84) × 10^6^ cells·cm^−2^, (4.79 ± 1.02) × 10^6^ cells·cm^−2^, (3.65 ± 0.45) × 10^6^ cells·cm^−2^, (3.50 ± 0.12) ×10^6^ cells·cm^−2^, and (4.74 ± 0.43) × 10^6^ cells·cm^−2^ in the control, DMSO control, acidification, BaP, and Acid + BaP combined treatment groups respectively. Levene’s test confirmed homogeneity of variances (*F*(4, 10)= 2.005, *p* = 0.170) and revealed a borderline significant difference among groups in cell density (one-way ANOVA, *F*(4, 10) = 3.488, *p* = 0.050; Kruskal–Wallis, H = 8.807, df = 4, *p* = 0.066), just above the conventional threshold for statistical significance. Compared with the control group, both single-stress treatments exhibited decreased cell densities, although the differences were not statistically the significant at the α = 0.05 level, whereas the combined treatment remained comparable to the control.

For chl.a content, the two-way ANOVA (Table 1) revealed a highly significant main effect of the treatment group (*F*(4, 20) = 8.055, *p* = 0.000, $\eta_{p}^{2}$ = 0.617), indicating substantial differences among groups. However, neither the main effect of time (*F*(1, 20) = 0.082, *p* = 0.778) nor the group * time interaction (*F*(4, 20) = 0.851, *p* = 0.510) reached statistical significance, suggesting that the temporal trends were largely consistent across groups. The model accounted for 47.9% of the total variance (adjusted *R^2^* = 0.479). Post hoc comparisons across pooled time points indicated that Acid, BaP and Acid + BaP groups had significatly lower content of chl.a than the control group (Table 1, *p* < 0.05). In addition, cell density had a significant main effect on treatment groups (*F*(4, 20) = 9.392, *p* = 0.000, $\eta_{p}^{2}$ = 0.653), as well as a main effect on time (*F*(1, 20) = 9.557, *p* = 0.006, $\eta_{p}^{2}$ = 0.323), while the group * time interaction (*F*(4, 20) = 0.982, *p* = 0.440) was not significant. The model accounted for 59.2% of the variance (adjusted *R^2^* = 0.592). When data from the two time points were combined for post hoc comparisons, cell density was found to be significantly reduced in Acid and BaP groups relative to the control group. At the same time, the Acid + BaP group showed no significant difference (Table 1, *p* > 0.05) from the control group.

### 3.2. Temporal Dynamics of Microbial Community Structure and Composition in Response to Treatment

Across all samples, a total of 124 Symbiodiniaceae lineages were identified, belongling to the genera *Cladocopium* and *Durusdinium*. *Cladocopium* was the dominant genus in all treatments, with subtype C3u accounting for more than 70% of the relative abundance. Additional subtypes included Cspc, C3d, C3w, C91, C115, C3b, C3aa, C119 and C1 (Figure S1). A total of 64 bacteria phyla and 165 bacteria classes were identified in coral-associated bacterial communities. The six most relative abundant bacteria phyla (Figure S3) were Proteobaceria (60.8–80.0%), Firmicutes (9.3–24.1%), Bacteroidota (1.3–11.6%), Actinobacteriota (1.1–9.1%), Acidobacteriota (0.07–1.6%) and Cyanobacteria (0.14–1.2%). The five most relative abundant bacteria classes were Gammaproteobacteria (25.4–51.3%), Alphaproteobacteria (13.3–42.1%), Bacilli (4.0–19.6%), Clostridia (1.7–6.5%) and Bacteroidia (1.5–6.0%). There were also six coral-associated archaea phyla identified in this study. Crenarchaeota (0–17.2%), Halobacterota (0–29.4%), Asgardarchaeota (0–2.8%), Thermoplasmatota (0–7.8%), Nanoarchaeota (0–8.9%) and Euryarchaeota (0–3.3%) were the most abundance archaeal phyla according to all the groups (Figure S2).

**Figure 2:**
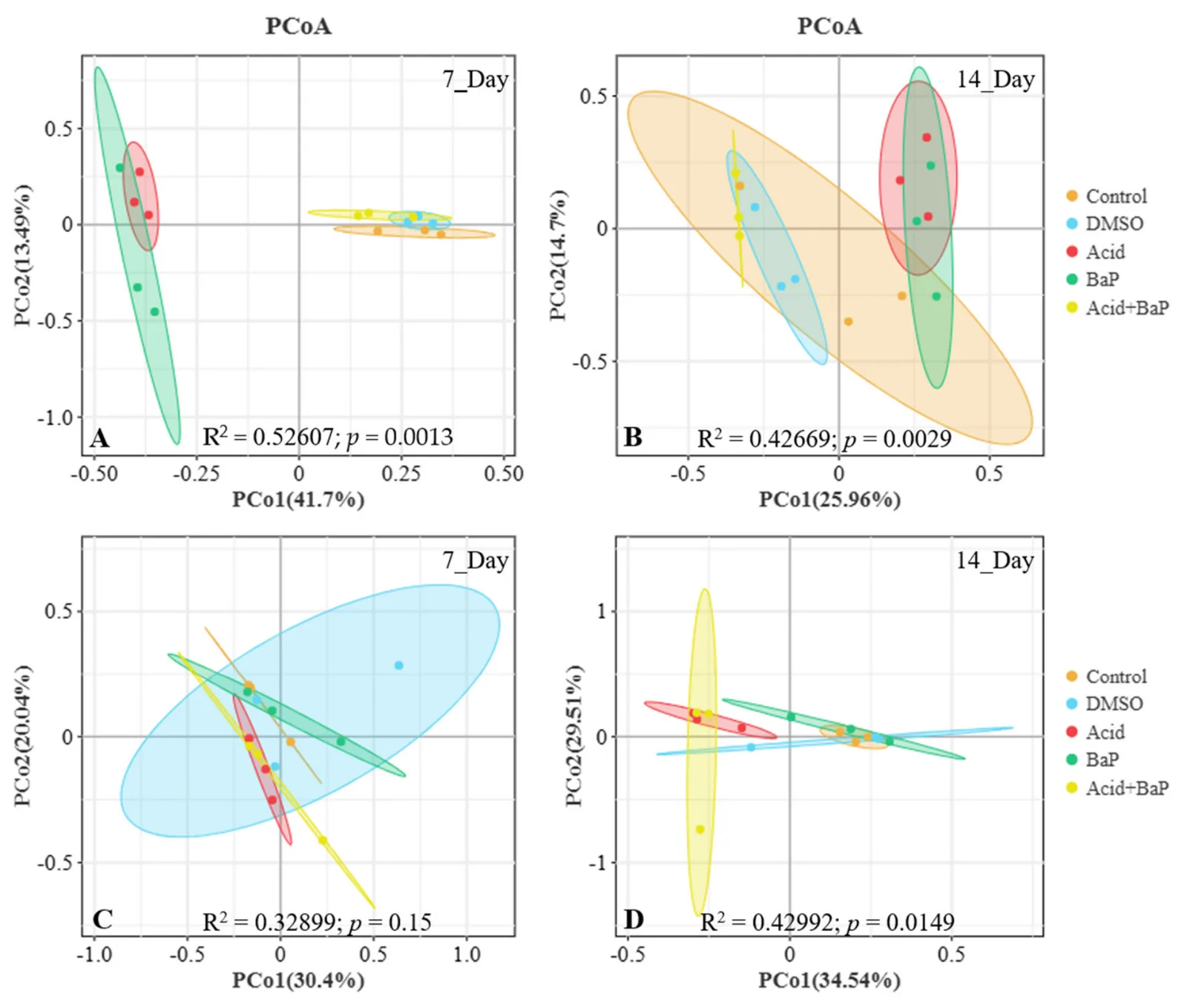
Principal Coordinate Analysis of coral-associated bacterial (**A**,**B**) and archaeal (**C**,**D**) community at the seventh and fourteenth day of exposure.

Principal Coordinate Analysis (PCoA), Analysis of Similarities (ANOSIM), and Permutational Multivariate Analysis of Variance (PERMANOVA) based on the Bray–Curtis distance showed dissimilarity and revealed distinct clustering patterns among treatments at each time point (Figure 2 and Figure S4). On day 7, this showed a clear separation in coral-associated bacterial communities among groups (PERMANOVA: R^2^ = 0.52607, *p* = 0.0013; ANOSIM: R = 0.3852, *p* = 0.0008). The first two principal coordinates explained 41.7% and 13.49% of the total variation, respectively (Figure 2A), while single-stress treatment clustering occurred closely together but were clearly separated from the control and combined-stress groups. Notably, the Acid + BaP group clustered closer to the control than to either single-stress group. By day 14, differences among bacterial community structures decreased but were still significant (PERMANOVA: R^2^ = 0.42669, *p* = 0.0029; ANOSIM: R = 0.3348, *p* = 0.0102), including partial convergence over time (Figure 2B). These results indicated that treatment identity explained a substantial proportion of the variation in community composition of bacterial communities. In contrast, coral-associated archaeal communities exhibited minimal separation among treatments on day 7 (Figure 2C), with no significant differences detected (PERMANOVA: R^2^ = 0.32899, *p* = 0.15; ANOSIM: R = 0.103, *p* = 0.1254). However, by day 14, significant differences in archaeal community composition were shown among groups (PERMANOVA: R^2^ = 0.42992, *p* = 0.0149; ANOSIM: R = 0.2585, *p* = 0.0121). Archaeal communities in the acidification and Acid + BaP treatments diverged markedly from the other groups, suggesting increased sensitivity of archaeal assemblages to prolonged acidification-related stress (Figure 2D). Interestingly, communities of coral-associated Symbiodiniaceae (Figure S4) were separated by groups on day 14 (PERMANOVA: R^2^ = 0.43021, *p* = 0.0159; ANOSIM: R = 0.2519, *p* = 0.0152) but intermixed on day 7 (PERMANOVA: R^2^ = 0.34793, *p* = 0.0814; ANOSIM: R = 0.09926, *p* = 0.1221).

When both time points were combined, treatment was the dominant factor shaping the community structure of coral-associated bacteria (single-factor: R^2^ = 0.28752, *p* = 0.0003; multi-factor: R^2^ = 0.28752, *p* = 0.0001). Time alone did not show a significant effect but became significant after accounting for treatment (Table S1), and a significant time * treatment interaction was detected, indicating that temporal variation was masked by strong treatment effects. Time had a strong effect on community composition of coral-associated archaea (single-factor: R^2^ = 0.0995, *p* = 0.0019; multi-factor: R^2^ = 0.0995, *p* = 0.0008). Treatment alone was not significant, but became significant after controlling for time (Table S1), and a significant time * treatment interaction was detected (multi-factor: R^2^ = 0.17778, *p* = 0.0157), indicating that time effects masked treatment variation in archaeal communities. PERMANOVA revealed significant effects of time (both single-factor and multi-factor rescults) in Symbiodiniaceae (Table S1).

**Figure 3:**
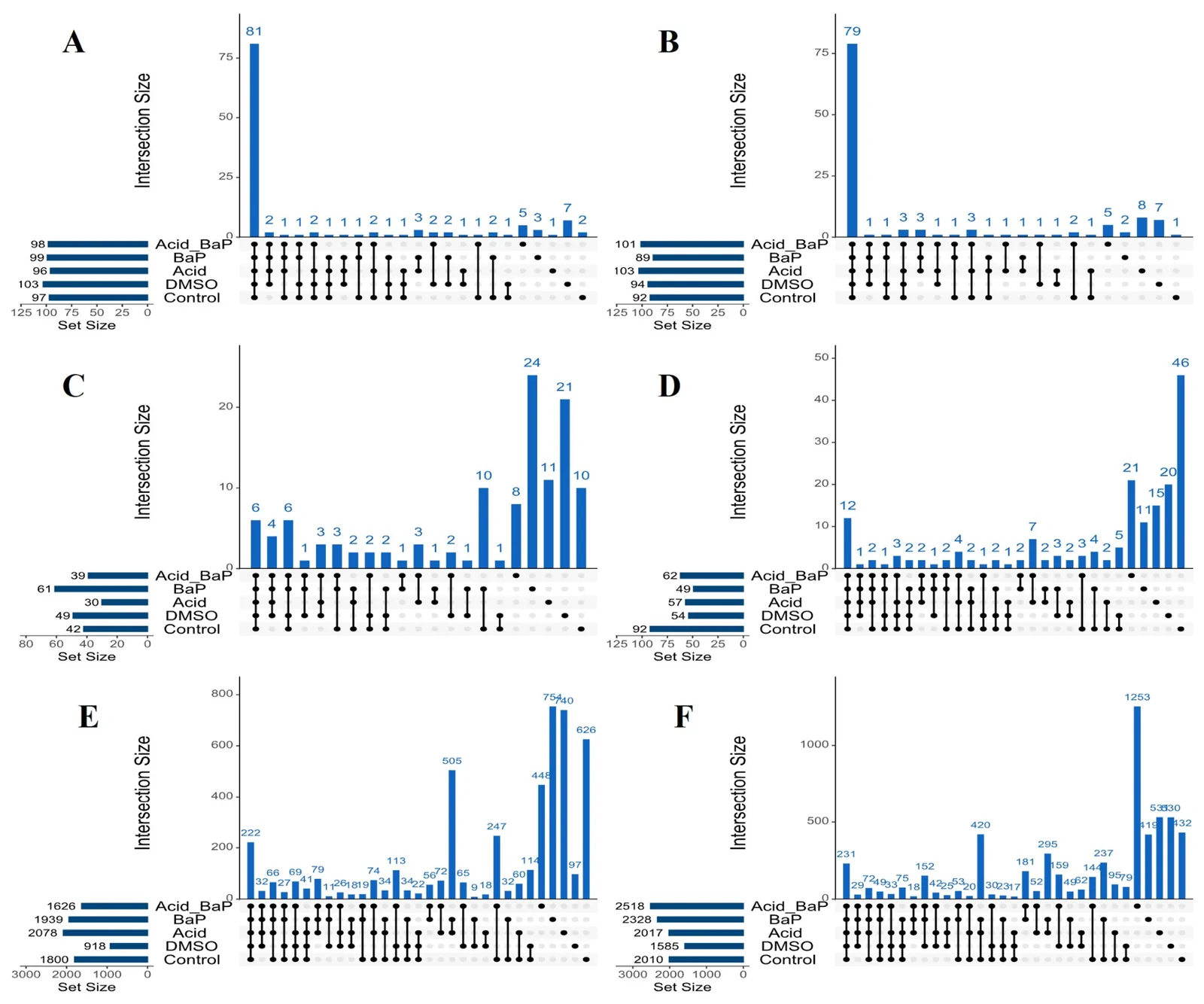
Composition of OTUs in all groups of Symbiodiniaceae (**A**,**B**), archaea (**C**,**D**) and bacteria (**E**,**F**). The seventh day of exposure including (**A**,**C**,**E**); the 14th day of exposure including (**B**,**D**,**F**).

Upset plot analysis based on the OTU level revealed 81 and 79 shared Symbiodiniaceae subtypes across all treatments on day 7 and 14, respectively. Compared with day 7, the acidification treatment exhibited a marked increase in unique Symbiodiniaceae subtypes by day 14 (Figure 3A,B). Most archaeal OTUs were unique to individual treatment. The number of OTUs shared exclusively among stress treatments increased from 5 to 13 on day 14 (Figure 3C,D), indicating time-dependent divergence under stress. For coral-associated bacteria, 222 and 231 OTUs were shared across all treatments on day 7 and 14, respectively. Similar to archaeal communities, a substantial proportion of bacterial OTUs were unique to specific treatments. Notably, single-stress treatments contained the highest number of unique OTUs on day 7, whereas the Acid + BaP combined treatment harbored the highest number of unique OTUs on day 14 (Figure 3E,F).

### 3.3. Core Microbial Communities Played a Dominated Role in Maintaining the Stability of Coral Internal Environment

**Figure 4:**
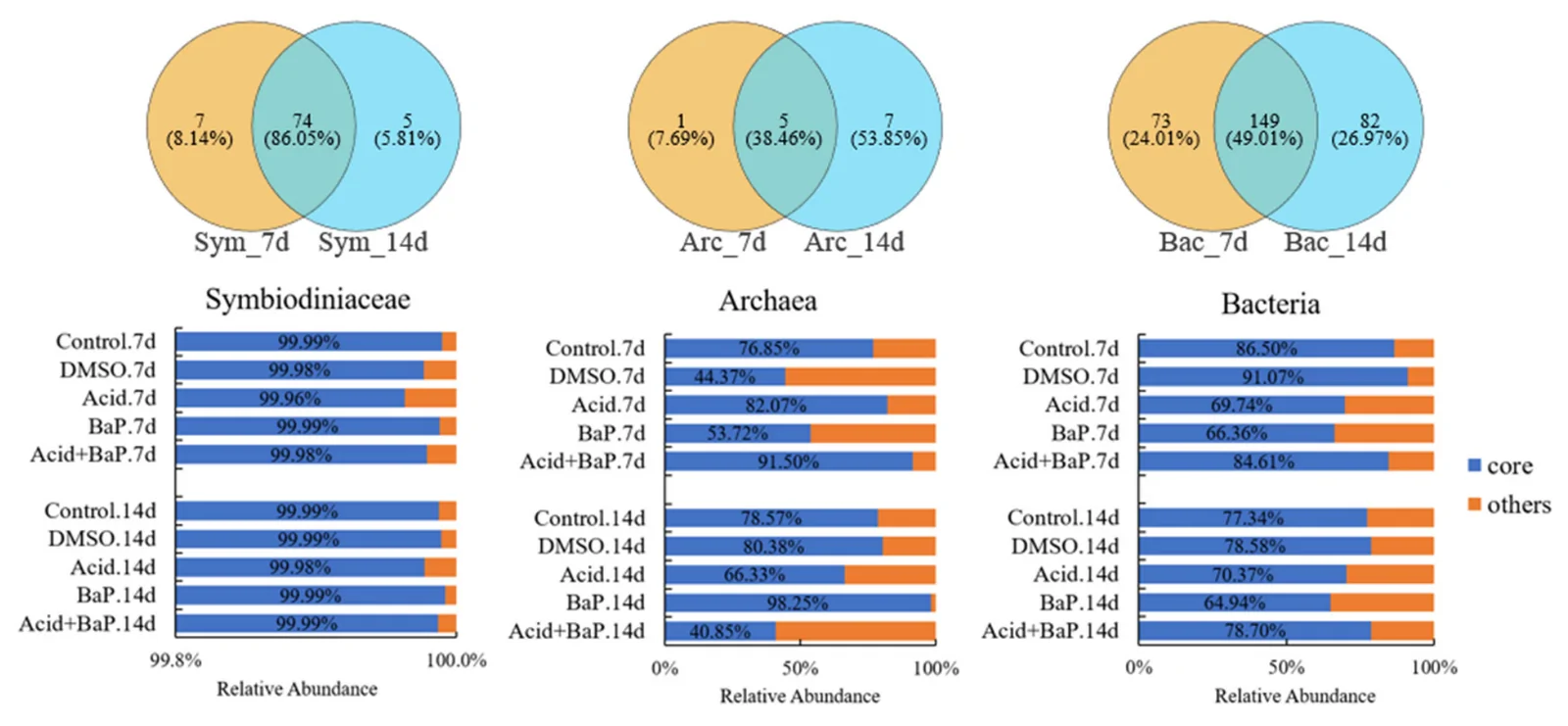
Relative abundance of core microbes in each group.

The number of shared microbial OTUs on day 7 and 14 is shown in Figure 3. Based on the Veen diagrams (Figure 4), OTUs shared between the two time points were first identified as a potential core microbiome. From this candidate pool, core taxa were operationally defined as those with a mean relative abundance > 1% and present in at least 50% of samples within each treatment group. In total, 74 Symbiodiniaceae, five archaeal, and 149 bacterial OTUs were identified as dominant microbial taxa. Notably, nearly all Symbiodiniaceae subtypes shared among different treatments at each exposure time point were also consistently shared between day 7 and 14, indicating a highly stable Symbiodiniaceae community structure.

Column charts illustrated the relative abundance of the potential core microbes across all treatment groups. Within the Symbiodiniaceae community, core subtypes accounted for more than 99% of the total relative abundance in all treatments at both exposure time points, demonstrating their overwhelming dominance. In contrast, the relative abundance patterns of coral-associated archaea appeared more variable. In the acidification and Acid + BaP combined treatment groups, the relative abundance of core archaeal OTUs increased on day 7 but declined on day 14, whereas the BaP treatment exhibited the opposite trend. Overall, control groups maintained similar dominant archaeal relative abundances across both exposure durations. In the bacterial community, dominant OTUs consistently accounted for more than 65% of the total relative abundance across all treatments and exposure times. However, a marked decline in the relative abundance of potential core bacterial OTUs was observed in the single-stress treatments (acidification and BaP) on day 7. In contrast, the Acid + BaP combined treatment consistently maintained a higher relative abundance of potential core bacterial OTUs than either single-stress treatment and was similar to the control groups (Figure 4).

### 3.4. The Effects of Acidification, BaP, and Combined Stress on Bacterial Dominant Core Taxa

**Figure 5:**
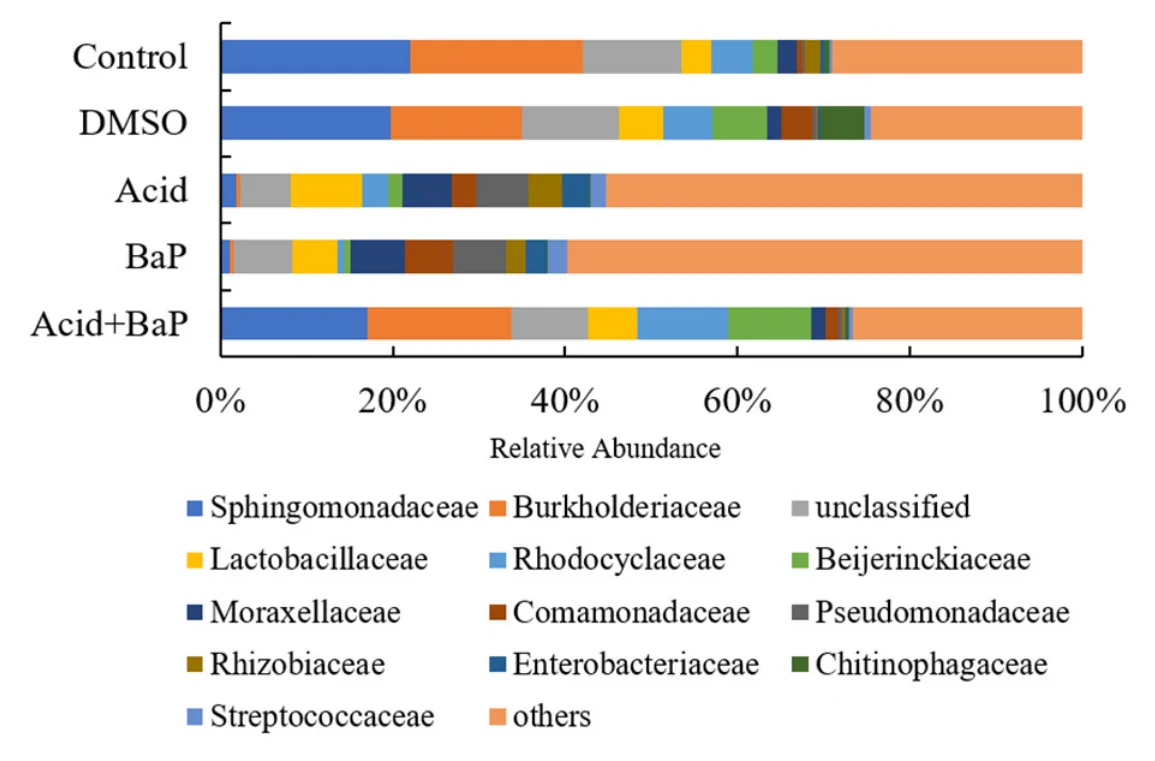
Relative abundances of core coral-associated bacterial (relative abundance > 1.0%) communities of all treatment groups.

The 149 dominant core coral-associated bacterial OTUs in Figure 4 were further classified, and their abundant taxonomic composition at the family level is shown in Figure 5 (relative abundance > 1%). The top ten relative abundances of core bacteria were Sphingomonadaceae (1.2–22.1%), Burkholderiaceae (0.43–20.0%), unclassified Proteobacteria (5.9–11.4%), Lactobacillaceae (3.4–8.3%), Rhodocyclaceae (0.8–10.6%), Beijerinckiaceae (0.8–9.5%), Moraxellaceae (1.6–6.3%), Comamonadaceae (0.7–5.6%), Pseudomonadaceae (0.2–6.2%), and Rhizobiaceae (0.1–3.9%). This showed that the composition structure of the core coral-associated bacteria had obvious changes in the single treatment groups, while the composition structure of Acid + BaP combined treatment group was similar to the control group, showing less influence from stress.

Bacterial OTUs with relative abundance ≥ 1% were classified as abundant taxa, while those ≤ 0.01% were defined as rare taxa. Figure 5 shows the relative abundance of abundant core bacteria changes among treatments, and one-way ANOVA revealed a significant difference (*F*(4, 25) = 6.262, *p* = 0.001). Compared with the control group, the relative abundance of acidification (Dunnett’s *t*-test, two-sided *p* = 0.039) and BaP (Dunnett’s *t*-test, two-sided *p* = 0.013) treatments significantly decreased, while the Acid + BaP treatment group had no significant difference. This trend generally corresponded to the changes in abundant bacteria among groups on the seventh day of stress (Figure S6).

## 4. Discussion

### 4.1. The Impact of Combined Stress on Physiological Indexes Was Less than That of Single Stress

Approximately 90% percent of the energy required for coral holobiont survival comes from Symbiodiniaceae through photosynthesis [32]. The growth and health of reef-building corals are closely related to the photosynthetic performance of their symbiotic dinoflagellates. Chlorophyll-a and Symbiodiniaceae cell density are therefore widely used as indicators of coral physiological status. In the present study, both chlorophyll-a content and Symbiodiniaceae cell density exhibited declining trends under all stress treatments, consistent with previous reports [12]. These results indicate that both single stressors (acidification or BaP) and combined stress (acidification + BaP) negatively affected symbiont cellular activity, further highlighting the high sensitivity of branching corals to environmental perturbations. Although synergistic effects have been reported for certain combined stressors, such as ocean acidification and warming [33,34], the mechanism underlying this synergy remains unclear [35].

Across both Kruskal–Wallis and PERMANOVA analyses, the combined-stress treatment consistently exhibited the largest *p* values relative to the control, except for the solvent control group, indicating that the chlorophyll-a content and cell diversity remained the most similar to the control. Even at 14 days, when overall group differences were no longer significant, these remained the closest to the control. This suggests that the combined-stress treatment had the least pronounced effect on chlorophyll-a and cell diversity, which tended to converge toward the control over time. Since the discrepancy between non-significant Dunn post hoc results and significant pairwise PERMANOVA may reflect differences in statistical sensitivity, the overall Kruskal–Wallis and PERMANOVA results both indicate significant treatment effects on chlorophyll-a and cell diversity on day 7, supporting the robustness of the main conclusion.

Although two-way ANOVA revealed highly significant main effects of both time and group, the time * group interaction was not significant (Table 1), indicating that the treatments followed consistent trends over time. After pooling the two time points, a significant difference from the control was observed in the single-stress group (Acid; BaP) but not in the combined-stress group (Acid + BaP), suggesting that the impact of combined stress on cell density of Symbiodiniaceae was less pronounced than that of single stress. Notably, when the interaction was not significant and the two time points were pooled, chlorophyll-a content was significantly reduced in the single-stress group BaP (mean difference = 6.47, *p* = 0.001) compared with the control, while the acidification (mean difference = 4.02, *p* = 0.050) and combined-stress groups (mean difference = 4.03, *p* = 0.049) showed *p* values near the 0.05 threshold, suggesting chlorophyll-a levels closer to those of the control than BaP group.

In contrast, our results demonstrated that the acidification–BaP combined treatment exerted a weaker effect on chlorophyll-a content and symbiont cell density than either single stressor. The deviation of observed from expected effects, detected at both exposure time points (7-day: obs > exp, *p* < 0.05; 14-day: obs > exp, *p* < 0.05; Δ = obs − exp exceeding the 95% confidence interval), together with the 95% confidence interval, suggests a possible antagonistic interaction between the two stressors, which contrasts with findings from other combined-stress studies [34,36]. However, because no formal interaction index based on the concentration addition or independent action model was calculated, this interpretation remains suggestive and requires further confirmation. The possible reasons for this potential antagonistic effect may be explained by several potential mechanisms that reduce BaP toxicity under acidified conditions. First, polycyclic aromatic hydrocarbon-degrading bacteria, including members Sphingomonadaceae and Pseudomonadaceae [37,38], constitute dominant components of the core microbial community of *Acropora millepora*. These bacteria exhibit optimal degradation activity at pH values between 7.0 and 8.0 [39], which overlaps with the experimental acidification conditions. Second, polyp or tentacle contraction under acidified seawater may reduce the coral surface area in contact with surrounding water, thereby limiting BaP accumulation. Additionally, Lactobacillaceae, recognized as beneficial core bacteria, can adsorb BaP [40], potentially reducing its bioavailability and uptake by the coral host. Since the potential antagonistic effect observed in this study was inferred from Symbiodiniaceae-related parameters (chlorophyll-a content and cell density), which reflect the state of the symbiotic partner rather than the coral host itself, the response of the coral polyp may differ. Therefore, we now describe this pattern as a potential antagonistic effect. This potential antagonistic effect was further supported by differences in coral-associated microbial community composition, where the combined-stress group remained more similar to the control than to either single-stress group.

### 4.2. Stability of Coral-Associated Symbiodiniaceae Maintains Coral Health

Compared with *Durusdinium*, *Cladocopium* exhibits higher photosynthetic efficiency [41,42] and stronger responsiveness to environmental fluctuations [43,44], and is also more widely distributed in tropical and subtropical coral reefs. Symbiodiniaceae community structure differed significantly among treatments on day 14 of exposure (Figure S4). Notably, PERMANOVA based on Bray–Curtis distances revealed a significant effect of time on community composition (R^2^ = 0.127, F = 4.048, *p* = 0.0097), whereas the main effect of treatment (R^2^ = 0.137, F = 1.093, *p* = 0.373) and the time * treatment interaction (R^2^ = 0.110, F = 0.878, *p* = 0.572) were not significant. These results indicate that community composition changed significantly over time, but no significant differences were detected among treatments, and the temporal trends were consistent across treatments. The non-significant treatment and interaction effects indicate that, under the present experimental conditions, the effect of treatment on Symbiodiniaceae was relatively weak or was masked by the stronger time effect. Possible explanations include insufficient treatment intensity, short treatment duration, strong resistance or resilience of the community, or high within-group variability masking of between-group differences. The temporal trends of community composition were similar across treatments. In addition, the residual R^2^ was 0.626, indicating that approximately 62.6% of community variation remained unexplained by time, treatment, or interaction. Future studies with increased replication and longer experimental duration are needed to further elucidate the drivers of community change.

C3u was identified as the dominant Symbiodiniaceae subtype, with relative abundances consistently exceeding others across all treatments (Figure S1). This subtype has also been reported as dominant in *Acropora latistella*, *Acropora digitifera*, *Hydnophora exesa* [45], and is considered relatively heat-resistant [46]. Stable associations between corals and subtype C3u have been documented in *Favites colemani* and *Acropora formosa* [47,48]. In our experiment, the Symbiodiniaceae community remained remarkably stable, with C3u maintaining absolute dominance under all stress conditions. Similar stability was observed by Tañedo et al., who reported persistent dominance of C3u in *Favites colemani* during a one-month exposure experiment, suggesting strong physiological adaptability of this symbiont to fluctuations in temperature and pH [47], and the symbiotic relationship of the dominant subtype was stable. Although corals dominated by C3u showed no significant decline in symbiont density after 72 h of heat stress [49], significant decreases were observed after 7 days of exposure to acidification or BaP in this study. While symbiont shuffling or acquisition has been reported in coral recruits under ocean warming and acidification [50], the persistence of a stable dominant Symbiodiniaceae community during early stress responses likely plays a crucial role in maintainting holobiont homeostasis.

### 4.3. Rapid Response of Coral-Associated Bacterial Community Facilitates Environmental Adaptation

**Figure 6:**
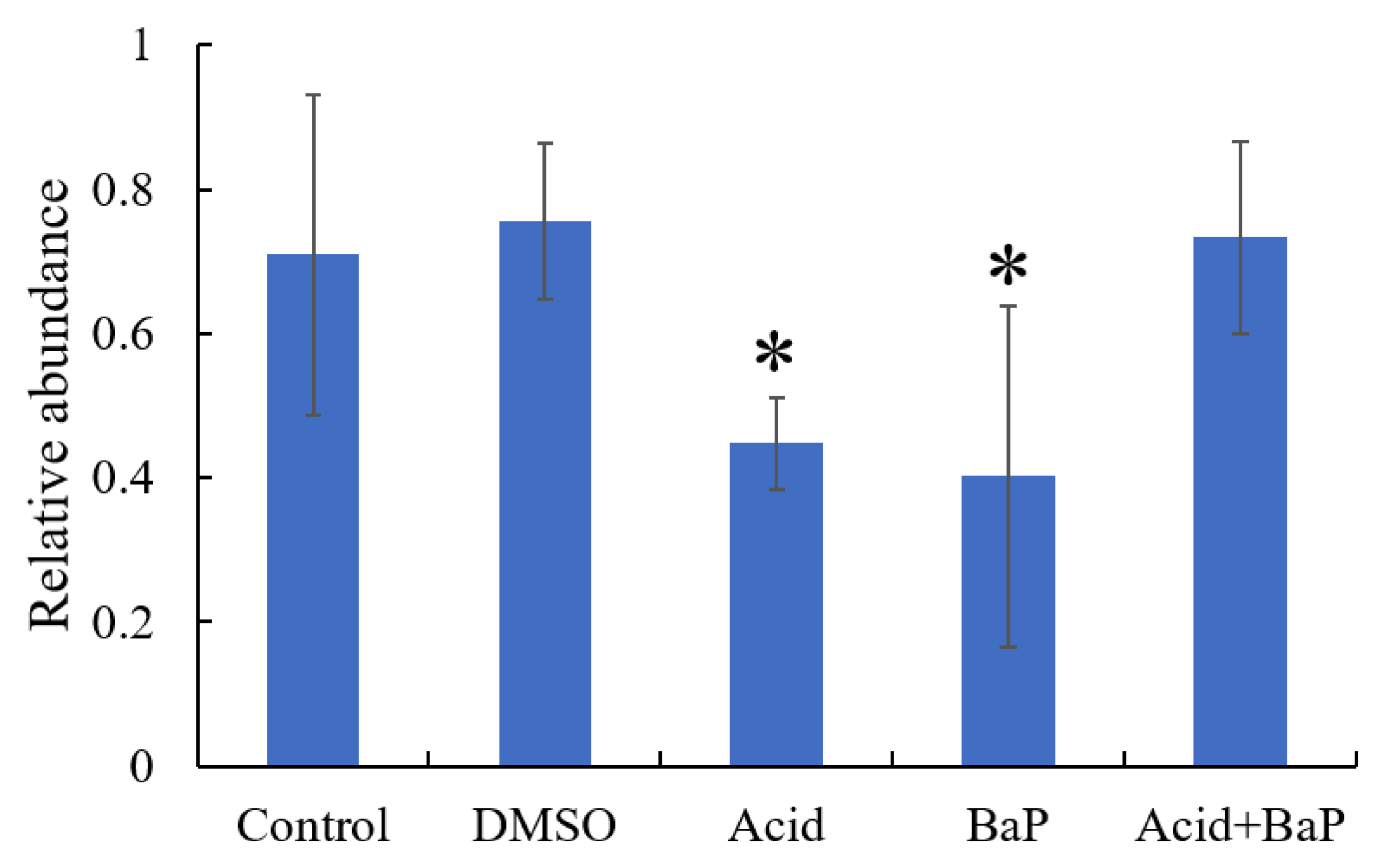
Different relative abundances of abundant coral-associated bacteria in different treatment groups. Error bars represent standard deviation (SD); “*” represented a significant difference (*p* < 0.05) compared with the control group.

Coral-associated microorganisms substantially enhance the ecological system and play a significant role in enhancing the environmental plasticity of coral holobionts. Compared with coral hosts, microbial communities have much shorter generation times and greater functional flexibility, enabling rapid responses to environmental stress [51]. Consistent with previous studies [14,52], the dominant bacterial phyla across treatments were Proteobacteria (60.8–80.0%), Firmicutes (9.3–24.1%), and Bacteroidetes (1.3–11.6%), while α-Proteobacteria and γ-Proteobacteria were the two classes with the highest relative abundance. The total relative abundance of core OTUs from Proteobacteria and Bacteroides decreased significantly in the acidification and the BaP treatment groups (Figure S7), possibly reflecting a reduction in beneficial bacterial taxa when community regulation is disrupted [53]. Although corals remained physiologically healthy, such shifts in core bacterial composition may increase vulnerability to long-term stress. Notably, no significant changes in core bacterial abundance were observed under combined stress (Figure 6).

Bacterial community structure differed significantly throughout the experiment period (Figure 2). PERMANOVA analysis results showed that treatment was identified as the primary driver of bacterial community composition (*p* = 0.0001), consistent with previous studies showing strong treatment effects. Interestingly, time alone did not significantly affect community structure (*p* = 0.1579), but became significant when treatment was included in the model (*p* = 0.0346). This suggests that temporal variation was masked by the dominant treatment effect, highlighting the importance of multi-factor models in detecting subtle temporal patterns. The significant time * treatment interaction (*p* = 0.0297) indicated that treatment effects varied over time, with a stronger effect at day 7 (Figure 2A, R^2^ = 0.52607) than at day 14 (Figure 2B, R^2^ = 0.42669). This temporal decline in treatment effect size may reflect community adaptation or convergence over time. Pairwise PERMANOVA further revealed that the bacterial community of the combined treatment group was significantly different from those of the single treatment groups, but did not differ significantly from the control or the solvent control (Figure S5). In contrast, both single treatment groups showed significant differences from the control. These results suggest that the interaction between the two stressors may have antagonistic or compensatory effects on the bacterial community, potentially buffering the community against the compositional shifts induced by each stressor alone. This finding provides community-level evidence consistent with the chlorophyll-a and cell diversity results, where the combined treatment group was also the most similar to the control. Together, these results support the interpretation that combined stress does not act as a simple sum of individual stressors, but instead leads to a distinct and less pronounced community response. Given the limited replication, these pairwise comparisons should be interpreted with caution.

As commonly observed in microbial ecosystems, coral-associated bacterial communities exhibited highly uneven abundance distributions [54]; abundant taxa comprised a small fraction of total OTUs, yet contributed disproportionately to overall bacterial abundance, whereas rare taxa accounted for a large proportion of OTUs but contributed minimally to total abundance. This pattern aligns with previous findings [55]. On day 7, the relative abundance of rare bacterial OTUs increased significantly in single-stress treatments (Figure S6), while abundant OTUs significantly declined in the acidification group (*p* < 0.05). Rare bacteria, despite their low abundance, represent a reservoir of genetic and functional diversity [56,57], and can provide critical ecological functions under environmental stress [58,59,60]. Importantly, single stressors exerted stronger impacts on the structure of core coral microbiomes under multifactorial stress conditions.

### 4.4. The Crucial Role of Core Microorganisms in Coral Holobionts

Sphingomonadaceae, recognized as efficient nitrogen-cycling and potential association-associated bacteria, are widely distributed in diverse natural environments, including extreme habitats, due to their metabolic versatility and ability to utilize a broad range of uncommon substrates [61,62]. In this study. Sphingomonadaceae were identified as a dominant core coral-associated bacterial family. Their well-documented capacity to degrade complex organic pollutants, particularly polycyclic aromatic hydrocarbons (PAHs), was reflected by significant variations in relative abundance across treatment groups. These features highlight their potential application in potential coral association strategies and their utility as biological indicators of coral health status.

Burkholderiaceae is another core coral-associated bacteria with *Acropora millepora*, which plays a multifaceted and crucial role in marine ecosystems. As a key participant in the carbon cycle, members of Burkholderiaceae can degrade complex organic compounds, including aromatic pollutants such as BaP, thereby linking organic carbon turnover with inorganic nutrient cycling and contributing to the overall metabolic balance of the coral holobiont [63,64]. However, the ecological role of Burkholderiaceae is dual in nature: while they possess strong potential associations, this family also includes opportunistic pathogens, such as members of the Burkholderia cepacia complex, which may pose pathogenic risks under unfavorable conditions [65,66]. Furthermore, the relative abundance of abundant dominant core bacterial taxa were significantly different among treatment groups (Table S2), and this may suggest that the stress treatments selectively reshaped the core microbial community. Notably, the combined treatment group showed a core taxa composition more similar to the control (Figure 5), consistent with the chlorophyll-a and cell diversity results.

Several nitrogen-cycling bacterial families—including Sphingomonadaceae, Rhizobiaceae, Bacillaceae, and Pseudomonadaceae [62,67]—were consistently present across all treatments, underscoring their essential roles in sustaining elemental cycling and metabolic stability within the coral holobiont. Lactobacillaceae, widely distributed in natural environments [68] and commonly recognized as beneficial commensal microbes and probiotics [69], showed their highest relative abundance under acidification stress. The less abundant but functionally important archaea were also an indispensable component of the coral functional holobiont. Most of the archaea shared in all groups were unknown, although they may play an important function role in symbiotic system and thus have significant gaps that need to be explored. In addition, core bacteria were prevalent in multiple ecological environments or hosts and used a wide range of ecological niches [62].

Several limitations should be noted in this study. The 14-day exposure may not capture longer-term dynamics; the single-location design limits generalizability; relative abundance data cannot provide absolute data changes; and functional and BaP accumulation measurements were not performed. However, the main focus of this study was the coral-associated microbial communities, and the coral polyp itself was not measured. But whether the potential antagonism observed in this study persists across holobiont components and over longer timescales remains an open and worthwhile question. Clarifying this relationship is important for understanding how coral holobionts respond to multiple co-occurring stressors in complex environments.

## 5. Conclusions

This study systematically characterized the responses of coral-associated microbial communities to single- (acidification or BaP) and combined-stress exposure, identifying microbial assemblages that contribute to the stabilization of coral holobiont homeostasis. Our findings provide a theoretical basis for the microbial prevention of coral bleaching, which had significant importance for coral restoration. A potential antagonistic interaction between the two stressors (ocean acidification and BaP pollution) remains suggestive and requires further confirmation, offering a novel perspective on how multiple environmental stressors jointly influence coral reef ecosystems. Based on the findings of this study, we suggest that, when facing environmental stresses potentially contributing to community redundancy and helping to maintain homeostasis within the coral holobiont, the coral-associated bacteria rapidly respond to the environment before other microorganisms, caused by changes in bacterial community structure, maintaining the composition of the core microbiome and increasing rare coral-associated bacteria communities to enhance similar community-level patterns. Contrary to the coral-associated bacteria, the structure of the Symbiodiniaceae community was relatively stable before environmental disturbances caused observable characteristics such as bleaching or lesions, and together with archaea, they maintain the energy and material metabolism of the corals to resist environmental changes. Overall, the dynamic equilibrium of microbial community composition and structure is maintained to sustain coral metabolism and survival.

## Figures and Tables

**Table 1:** Two-way ANOVA and post hoc comparison results of physiological indexes.

	Values Presented as Mean ± SD					*p* Value		
	Control	DMSO	Acid	BaP	Acid + BaP	Group	Time	Group * Time
Chl-a (µg·cm^−2^)	16.63 ± 2.40 ^a^	16.05 ± 2.02 ^ab^	12.60 ± 3.03 ^bc^	10.16 ± 1.95 ^c^	12.59 ± 1.62 ^bc^	0.000	0.778	0.510
Cell density (cells·cm^−2^)	(4.92 ± 0.76) × 10^6 a^	(4.65 ± 0.86) × 10^6 a^	(3.14 ± 0.68) × 10^6 b^	(3.11± 0.50) × 10^6 b^	(3.96± 1.04) × 10^6 ab^	0.000	0.006	0.440

Note: Different superscript letters (^a^, ^b^, ^c^) indicate significant differences between groups (*p* < 0.05, Tukey’s HSD post hoc test).

## Data Availability

The original contributions presented in this study are included in the article/Supplementary Material. Further inquiries can be directed to the corresponding authors.

## Supplementary Materials

The following supporting information can be downloaded at: https://www.mdpi.com/article/10.3390/microorganisms14102259/s1, Figure S1. The relative abundance of the top ten coral-associated Symbiodiniaceae in different treatment groups. Figure S2. The relative abundance of the top ten coral-associated archaea in different treatment groups at class level. Figure S3. The relative abundance of the top ten coral-associated bacteria in different treatment groups at class level. Figure S4. Principal component analysis of Symbiodiniaceae community at the seventh (left) and fourteenth (right) day of exposure. Figure S5. Matrix of pairwise PERMANOVA P-values for treatment groups of coral-associated bacteria. Figure S6. Differences in the relative abundance of abundant, rare and moderate of coral-associated bacteria in different treatment groups. Figure S7. Comparison of OTUs relative abundance ratio at phylum level in different treatment groups. Table S1. PERMANOVA analysis results of microbial communitiesthe from combined dataset of the two time points based on Bray-Curtis distances. Table S2. Comparative changes in the relative abundance of abundant dominant core bacterial taxa relative to the control group.

## References

[1] Hughes D.J., Alderdice R., Cooney C., Kuehl M., Pernice M., Voolstra C.R., Suggett D.J. (2020). Coral Reef Survival under Accelerating Ocean Deoxygenation. Nat. Clim. Change.

[2] Voolstra C.R., Peixoto R.S., Ferrier-Pages C. (2023). Mitigating the Ecological Collapse of Coral Reef Ecosystems Effective Strategies to Preserve Coral Reef Ecosystems. EMBO Rep..

[3] Virgen-Urcelay A., Donner S.D. (2023). Increase in the Extent of Mass Coral Bleaching over the Past Half-Century, Based on an Updated Global Database. PLoS ONE.

[4] Blackall L.L., Wilson B., van Oppen M.J.H. (2015). Coral-the World’s Most Diverse Symbiotic Ecosystem. Mol. Ecol..

[5] LaJeunesse T.C., Parkinson J.E., Gabrielson P.W., Jeong H.J., Reimer J.D., Voolstra C.R., Santos S.R. (2018). Systematic Revision of Symbiodiniaceae Highlights the Antiquity and Diversity of Coral Endosymbionts. Curr. Biol..

[6] Bourne D., Iida Y., Uthicke S., Smith-Keune C. (2008). Changes in Coral-Associated Microbial Communities during a Bleaching Event. ISME J..

[7] Hsiao S.-C., Wu H.-L., Fu H.-S., Chen W.-B. (2025). Accelerated Ocean Acidification (1985–2022) Threatens Tropical Coral Reefs and Highlights Biogeochemical Refugia for Marine Conservation. J. Sea Res..

[8] Ashina C., Pugazhenthiran N., Mangalaraja R.V., Sathishkumar P. (2025). Review on Enhancing Solar Photocatalysis for Sustainable Degradation of Invisible Environmental Pollutants. Renew. Sustain. Energy Rev..

[9] Meron D., Rodolfo-Metalpa R., Cunning R., Baker A.C., Fine M., Banin E. (2012). Changes in Coral Microbial Communities in Response to a Natural pH Gradient. ISME J..

[10] Bindoff N.L., Cheung W.W.L., Kairo J.G., Arístegui J., Guinder V.A., Hallberg R., Hilmi N., Jiao N., O’Donoghue S., Acar S. (2022). Changing Ocean, Marine Ecosystems, and Dependent Communities. The Ocean and Cryosphere in a Changing Climate: Special Report of the Intergovernmental Panel on Climate Change.

[11] Xu Q., Wang Q., Ma R., Cai R., Xie S., Miao J., Pan L. (2026). Metabolic Toxicity of PAHs in Ruditapes Philippinarum: Metabolic Pathways and Hepatotoxicity. Aquat. Toxicol..

[12] Xiang N., Jiang C., Huang W., Nordhaus I., Zhou H., Drews M., Diao X. (2019). The Impact of Acute Benzo(a)Pyrene on Antioxidant Enzyme and Stress-Related Genes in Tropical Stony Corals (*Acropora* spp.). Sci. Total Environ..

[13] Anthony K.R.N., Kline D.I., Diaz-Pulido G., Dove S., Hoegh-Guldberg O. (2008). Ocean Acidification Causes Bleaching and Productivity Loss in Coral Reef Builders. Proc. Natl. Acad. Sci. USA.

[14] Ge R., Liang J., Yu K., Chen B., Yu X., Deng C., Chen J., Xu Y., Qin L. (2021). Regulation of the Coral-Associated Bacteria and Symbiodiniaceae in *Acropora valida* Under Ocean Acidification. Front. Microbiol..

[15] Webster N.S., Negri A.P., Botte E.S., Laffy P.W., Flores F., Noonan S., Schmidt C., Uthicke S. (2016). Host-Associated Coral Reef Microbes Respond to the Cumulative Pressures of Ocean Warming and Ocean Acidification. Sci. Rep..

[16] Bourne D.G., Morrow K.M., Webster N.S. (2016). Insights into the Coral Microbiome: Underpinning the Health and Resilience of Reef Ecosystems. Annu. Rev. Microbiol..

[17] Mason R.A.B. (2018). Decline in Symbiont Densities of Tropical and Subtropical Scleractinian Corals under Ocean Acidification. Coral Reefs.

[18] Grottoli A.G., Martins P.D., Wilkins M.J., Johnston M.D., Warner M.E., Cai W.-J., Melman T.F., Hoadley K.D., Pettay D.T., Levas S. (2018). Coral Physiology and Microbiome Dynamics under Combined Warming and Ocean Acidification. PLoS ONE.

[19] Hu L., Shi X., Qiao S., Lin T., Li Y., Bai Y., Wu B., Liu S., Kornkanitnan N., Khokiattiwong S. (2017). Sources and Mass Inventory of Sedimentary Polycyclic Aromatic Hydrocarbons in the Gulf of Thailand: Implications for Pathways and Energy Structure in SE Asia. Sci. Total Environ..

[20] Xiang N., Jiang C., Yang T., Li P., Wang H., Xie Y., Li S., Zhou H., Diao X. (2018). Occurrence and Distribution of Polycyclic Aromatic Hydrocarbons (PAHs) in Seawater, Sediments and Corals from Hainan Island, China. Ecotoxicol. Environ. Saf..

[21] Yang T., Diao X., Cheng H., Wang H., Zhou H., Zhao H., Chen C.M. (2020). Comparative Study of Polycyclic Aromatic Hydrocarbons (PAHs) and Heavy Metals (HMs) in Corals, Sediments and Seawater from Coral Reefs of Hainan, China. Environ. Pollut..

[22] Nikinmaa M. (2013). Climate Change and Ocean Acidification Interactions with Aquatic Toxicology. Aquat. Toxicol..

[23] Han M., Zhang R., Yu K., Li A., Wang Y., Huang X. (2020). Polycyclic Aromatic Hydrocarbons (PAHs) in Corals of the South China Sea: Occurrence, Distribution, Bioaccumulation, and Considerable Role of Coral Mucus. J. Hazard. Mater..

[24] Chen Y., Qi Z., Yin L., Chang F., Ju H., Jing H., Diao X. (2026). Multi-Level Holobiont Dysregulation Increases the Ecological Risk of Combined Ocean Acidification and Benzo[a]Pyrene Pollution to the Reef-Building Coral Porites Lutea. J. Hazard. Mater..

[25] Jeffrey S.W., Humphrey G.F. (1975). New Spectrophotometric Equations for Determining Chlorophylls *a*, *b*, *c*1 and *c*2 in Higher Plants, Algae and Natural Phytoplankton. Biochem. Physiol. Pflanz..

[26] Stat M., Pochon X., Cowie R., Gates R. (2009). Specificity in Communities of Symbiodinium in Corals from Johnston Atoll. Mar. Ecol. Prog. Ser..

[27] Yang T., Qi Z., Wang H., Zheng P., Kao S.-J., Diao X. (2025). Seasonal Variation in In Hospite but Not Free-Living, Symbiodiniaceae Communities Around Hainan Island, China. Microorganisms.

[28] Zhang Y., Yang Q., Zhang Y., Ahmad M., Ling J., Tang X., Dong J. (2021). Shifts in Abundance and Network Complexity of Coral Bacteria in Response to Elevated Ammonium Stress. Sci. Total Environ..

[29] Gantner S., Andersson A.F., Alonso-Saez L., Bertilsson S. (2011). Novel Primers for 16S rRNA-Based Archaeal Community Analyses in Environmental Samples. J. Microbiol. Methods.

[30] Caporaso J.G., Kuczynski J., Stombaugh J., Bittinger K., Bushman F.D., Costello E.K., Fierer N., Pena A.G., Goodrich J.K., Gordon J.I. (2010). QIIME Allows Analysis of High-Throughput Community Sequencing Data. Nat. Methods.

[31] Quast C., Pruesse E., Yilmaz P., Gerken J., Schweer T., Yarza P., Peplies J., Gloeckner F.O. (2013). The SILVA Ribosomal RNA Gene Database Project: Improved Data Processing and Web-Based Tools. Nucleic Acids Res..

[32] Zhao M.X., Yu K.F., Zhang Q.M., Shi Q., Roff G. (2014). Age Structure of Massive *Porites lutea* Corals at Luhuitou Fringing Reef (Northern South China Sea) Indicates Recovery Following Severe Anthropogenic Disturbance. Coral Reefs.

[33] Carbonne C., Comeau S., Chan P.T.W., Plichon K., Gattuso J.-P., Teixido N. (2022). Early Life Stages of a Mediterranean Coral Are Vulnerable to Ocean Warming and Acidification. Biogeosciences.

[34] Prada F., Caroselli E., Mengoli S., Brizi L., Fantazzini P., Capaccioni B., Pasquini L., Fabricius K.E., Dubinsky Z., Falini G. (2017). Ocean Warming and Acidification Synergistically Increase Coral Mortality. Sci. Rep..

[35] Van der Zande R.M., Achlatis M., Bender-Champ D., Kubicek A., Dove S., Hoegh-Guldberg O. (2020). Paradise Lost: End-of-Century Warming and Acidification under Business-as-Usual Emissions Have Severe Consequences for Symbiotic Corals. Glob. Change Biol..

[36] Chen Y.-T., Ding D.-S., Lim Y.C., Dong C.-D., Hsieh S.-L. (2024). Combined Toxicity of Microplastics and Copper on *Goniopora columns*. Environ. Pollut..

[37] Waigi M.G., Sun K., Gao Y. (2017). Sphingomonads in Microbe-Assisted Phytoremediation: Tackling Soil Pollution. Trends Biotechnol..

[38] Sun Y., Ding A., Zhao X., Chang W., Ren L., Zhao Y., Song Z., Hao D., Liu Y., Jin N. (2022). Response of Soil Microbial Communities to Petroleum Hydrocarbons at a Multi-Contaminated Industrial Site in Lanzhou, China. Chemosphere.

[39] Muangchinda C., Yamazoe A., Polrit D., Thoetkiattikul H., Mhuantong W., Champreda V., Pinyakong O. (2017). Biodegradation of High Concentrations of Mixed Polycyclic Aromatic Hydrocarbons by Indigenous Bacteria from a River Sediment: A Microcosm Study and Bacterial Community Analysis. Environ. Sci. Pollut. Res..

[40] Attia H., ElBanna S.A., Khattab R.A., Farag M.A., Yassin A.S., Aziz R.K. (2023). Integrating Microbiome Analysis, Metabolomics, Bioinformatics, and Histopathology to Elucidate the Protective Effects of Pomegranate Juice against Benzo-Alpha-Pyrene-Induced Colon Pathologies. Int. J. Mol. Sci..

[41] Chen B., Yu K., Qin Z., Liang J., Wang G., Huang X., Wu Q., Jiang L. (2020). Dispersal, Genetic Variation, and Symbiont Interaction Network of Heat-Tolerant Endosymbiont *Durusdinium trenchii*: Insights into the Adaptive Potential of Coral to Climate Change. Sci. Total Environ..

[42] Baker D.M., Andras J.P., Jordan-Garza A.G., Fogel M.L. (2013). Nitrate Competition in a Coral Symbiosis Varies with Temperature among Symbiodinium Clades. ISME J..

[43] Simon J.-C., Marchesi J.R., Mougel C., Selosse M.-A. (2019). Host-Microbiota Interactions: From Holobiont Theory to Analysis. Microbiome.

[44] Yuyama I., Nakamura T., Higuchi T., Hidaka M. (2016). Different Stress Tolerances of Juveniles of the Coral *Acropora tenuis* Associated with Clades C1 and D Symbiodinium. Zool. Stud..

[45] Huang Y., Feng C., Gao B., Long L., Yang F. (2025). Seasonal Dynamics of Symbiodiniaceae Communities Associated with Nine Coral Species in Luhuitou Coral Reef, South China Sea. Acta Oceanol. Sin..

[46] Chen B., Wei Y., Yu K., Liang Y., Yu X., Liao Z., Qin Z., Xu L., Bao Z. (2024). The Microbiome Dynamics and Interaction of Endosymbiotic Symbiodiniaceae and Fungi Are Associated with Thermal Bleaching Susceptibility of Coral Holobionts. Appl. Environ. Microbiol..

[47] Tanedo M.C.S., Villanueva R.D., Torres A.F., Ravago-Gotanco R., Lourdes San Diego-McGlone M. (2021). Individual and Interactive Effects of Ocean Warming and Acidification on Adult Favites Colemani. Front. Mar. Sci..

[48] Amid C., Olstedt M., Gunnarsson J.S., Le Lan H., Tran Thi Minh H., Van den Brink P.J., Hellstrom M., Tedengren M. (2018). Additive Effects of the Herbicide Glyphosate and Elevated Temperature on the Branched Coral *Acropora formosa* in Nha Trang, Vietnam. Environ. Sci. Pollut. Res..

[49] Da-Anoy J.P., Cabaitan P.C., Conaco C. (2019). Species Variability in the Response to Elevated Temperature of Select Corals in North-Western Philippines. J. Mar. Biol. Assoc. UK.

[50] Fabina N.S., Putnam H.M., Franklin E.C., Stat M., Gates R.D. (2013). Symbiotic Specificity, Association Patterns, and Function Determine Community Responses to Global Changes: Defining Critical Research Areas for Coral-Symbiodinium Symbioses. Glob. Change Biol..

[51] Vanwonterghem I., Webster N.S. (2020). Coral Reef Microorganisms in a Changing Climate. iScience.

[52] Krishnaswamy V.G., Mani K., Kumar P.S., Rangasamy G., Sridharan R., Rethnaraj C., Ganesh S.S.A., Kalidas S., Palanisamy V., Chellama N.J. (2023). Prevalence of Differential Microbiome in Healthy, Diseased and Nipped Colonies of Corals, *Porites lutea* in the Gulf of Kachchh, North-West Coast of India. Environ. Res..

[53] Zhu W., Liu X., Zhu M., Li X., Yin H., Huang J., Wang A., Li X. (2022). Responses of Symbiodiniaceae Shuffling and Microbial Community Assembly in Thermally Stressed *Acropora hyacinthus*. Front. Microbiol..

[54] Jousset A., Bienhold C., Chatzinotas A., Gallien L., Gobet A., Kurm V., Kuesel K., Rillig M.C., Rivett D.W., Salles J.F. (2017). Where Less May Be More: How the Rare Biosphere Pulls Ecosystems Strings. ISME J..

[55] Qi Z., Diao X., Yang T., Zeng R., Wang H., Zhou H. (2022). Spatial and Interspecific Differences in Coral-Associated Bacterial Diversity in Hainan, China. Mar. Pollut. Bull..

[56] Wang H., Qi Z., Zheng P., Jiang C., Diao X. (2022). Abundant and Rare Microbiota Assembly and Driving Factors between Mangrove and Intertidal Mudflats. Appl. Soil Ecol..

[57] Liang Y., Xiao X., Nuccio E.E., Yuan M., Zhang N., Xue K., Cohan F.M., Zhou J., Sun B. (2020). Differentiation Strategies of Soil Rare and Abundant Microbial Taxa in Response to Changing Climatic Regimes. Environ. Microbiol..

[58] Che W., Tan X., Zhao H., Man Y. (2025). Comparison of the Environmental Adaptation Strategies of Abundant and Rare Taxa in the Sediments of the Northwestern South China Sea. J. Geophys. Res. Ocean..

[59] Sauret C., Severin T., Vetion G., Guigue C., Goutx M., Pujo-Pay M., Conan P., Fagervold S.K., Ghiglione J.-F. (2014). “Rare Biosphere” Bacteria as Key Phenanthrene Degraders in Coastal Seawaters. Environ. Pollut..

[60] Zhang W., Wang X., Miao Y., Li Y., Zhang H., Niu L., Wang L. (2021). Determining the Effect of Sertraline on Nitrogen Transformation through the Microbial Food Web in Sediments Based on 15N-DNA-Stable Isotope Probing. Environ. Res..

[61] Arkan-Ozdemir S., Üstüntürk-Onan M., Ilhan-Sungur E. (2025). *Facivitalis istanbulensis* Gen. Nov., sp. Nov., a Novel Member of the Family *Sphingomonadaceae* with the Potential for Aromatic-Degradation Isolated from Jet A1 Fuel. Antonie Van Leeuwenhoek.

[62] Yang Y., Xu N., Zhang Z., Lei C., Chen B., Qin G., Qiu D., Lu T., Qian H. (2024). Deciphering Microbial Community and Nitrogen Fixation in the Legume Rhizosphere. J. Agric. Food Chem..

[63] Fan L., Liu M., Simister R., Webster N.S., Thomas T. (2013). Marine Microbial Symbiosis Heats up: The Phylogenetic and Functional Response of a Sponge Holobiont to Thermal Stress. ISME J..

[64] Thomas T., Moitinho-Silva L., Lurgi M., Bjoerk J.R., Easson C., Astudillo-Garcia C., Olson J.B., Erwin P.M., Lopez-Legentil S., Luter H. (2016). Diversity, Structure and Convergent Evolution of the Global Sponge Microbiome. Nat. Commun..

[65] Terzin M., Robbins S.J., Bell S.C., Cao K.-A.L., Gruber R.K., Frade P.R., Webster N.S., Yeoh Y.K., Bourne D.G., Laffy P.W. (2025). Gene Content of Seawater Microbes Is a Strong Predictor of Water Chemistry across the Great Barrier Reef. Microbiome.

[66] Engelberts J.P., Robbins S.J., de Goeij J.M., Aranda M., Bell S.C., Webster N.S. (2020). Characterization of a Sponge Microbiome Using an Integrative Genome-Centric Approach. ISME J..

[67] Zang H., Shi W., Kau M., Li J., Li J., Zhang W., Zhou Z., Sun B., Yuan L., Zhu R. (2025). Effects of Elevated CO_2_ Concentration on Se Accumulation and Associated Rhizobacterial Community in *Cardamine hupingshanensis*. Plant Soil.

[68] Kullar R., Goldstein E.J.C., Johnson S., McFarland L.V. (2023). *Lactobacillus* Bacteremia and Probiotics: A Review. Microorganisms.

[69] Du R., Han F., Li Z., Yu J., Xu Y., Huang Y., Wu Q. (2025). Uncovering Encrypted Antimicrobial Peptides in Health-Associated *Lactobacillaceae* by Large-Scale Genomics and Machine Learning. Microbiome.

