# Supplementary material for: Transient Disturbance and Community Convergence of Coral-Associated Microbial Communities Under Acidification, Benzo[a]pyrene Pollution, and Combined Stress in Coral Acropora millepora
Source: Microorganisms. 2026 Oct 4;14(10):2259. doi: 10.3390/microorganisms14102259 (PMC13707767; doi:10.3390/microorganisms14102259)
Supplement: Supplementary file 1 [file microorganisms-14-02259-s001.zip › microorganisms-4509158-supplementary.pdf]

Supplementary file:

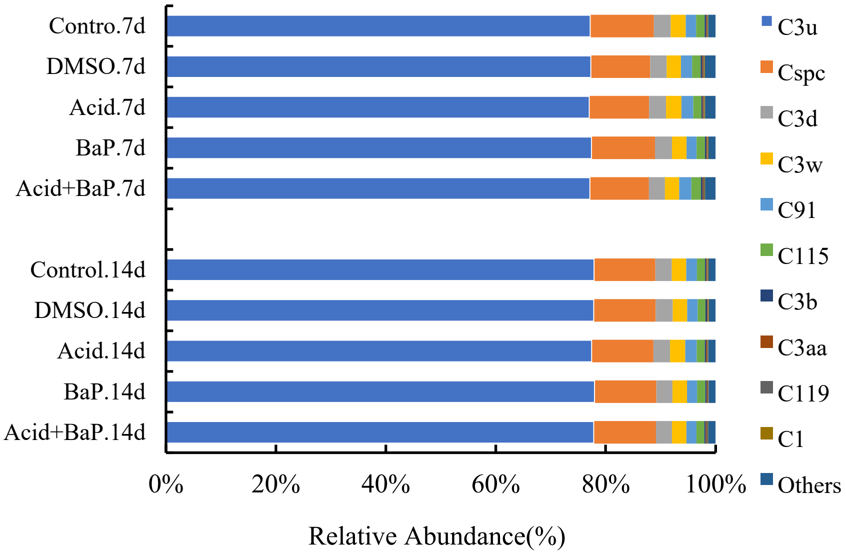

Figure S1. The relative abundance of the top ten coral-associated Symbiodiniaceae in different treatment groups. Control, Control group; DMSO, Solvent control group; Acid, Acidification treatment group; BaP, BaP treatment group; Acid+BaP, Combined treatment group; 7 d means the seventh day of exposure; 14 d means the fourteenth day of exposure.

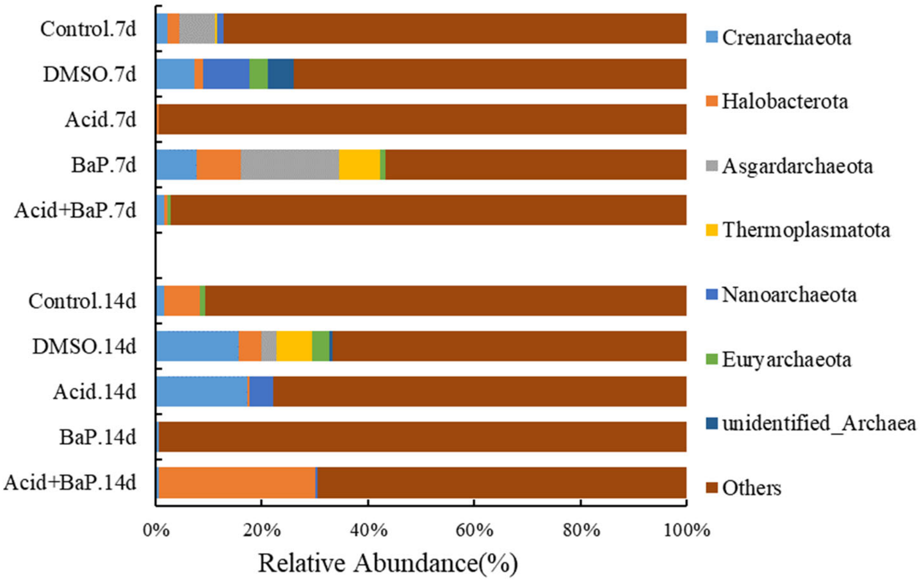

Figure S2. The relative abundance of the coral-associated archaea in different treatment groups at plylum level. Control, Control group; DMSO, Solvent control group; Acid, Acidification treatment group; BaP, BaP treatment group; Acid+BaP, Combined treatment group; 7 d means the seventh day of exposure; 14 d means the fourteenth day of exposure.

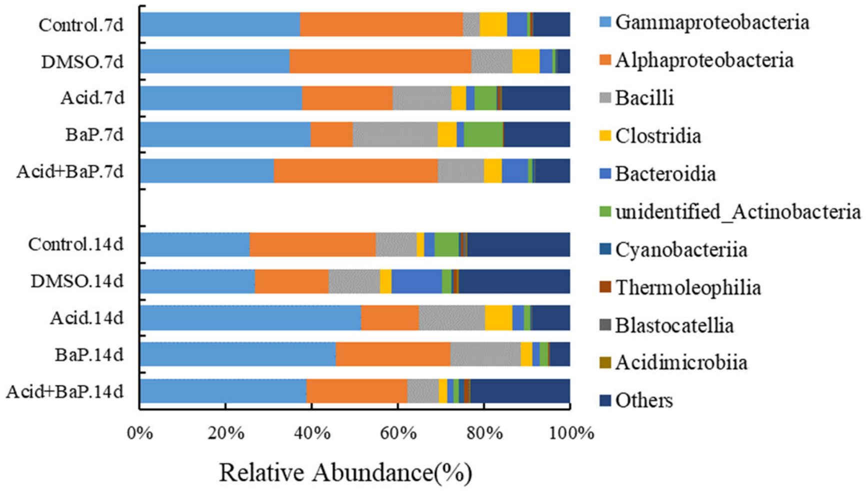

Figure S3. The relative abundance of the top ten coral-associated bacteria in different treatment groups at class level. Control, Control group; DMSO, Solvent control group; Acid, Acidification treatment group; BaP, BaP treatment group; Acid+BaP, Combined treatment group; 7 d means the seventh day of exposure; 14 d means the fourteenth day of exposure.

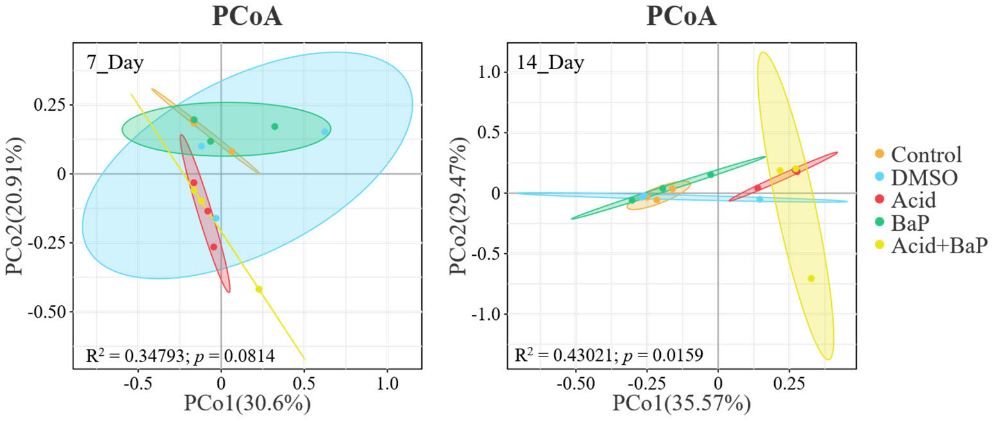

Figure S4. Principal Coordinates Analysis of Symbiodiniaceae community at the seventh (left) and fourteenth (right) day of exposure.

Table S1. PERMANOVA analysis results of microbial communitiesthe from combined dataset of the two time points based on Bray-Curtis distances .

Sample	Analysis	Source	Df	R^2	F	P
Bacteria	Single-fator	time	1	0.05331	1.5767	0.1047
		treatment	4	0.28752	2.5222	**0.0003**
	Two-factor	time	1	0.05331	2.1378	**0.0346**
		treatment	4	0.28752	2.8826	**0.0001**
		time*treatment	4	0.16045	1.6087	**0.0297**
Archaea	Single-fator	time	1	0.0995	3.094	**0.0019**
		treatment	4	0.16332	1.22	0.1506
	Two-factor	time	1	0.0995	3.5576	**0.0008**
		treatment	4	0.16332	1.4598	**0.0348**
		time*treatment	4	0.17778	1.5891	**0.0157**
Symbiodiniaceae	Single-fator	time	1	0.12676	4.0646	**0.0065**
		treatment	4	0.13686	0.991	0.4555
	Two-factor	time	1	0.12676	4.0476	**0.0097**
		treatment	4	0.13686	1.0925	0.3726
		time*treatment	4	0.11002	0.8783	0.5718

Abbreviations: Df, degrees of freedom; sum of squares; R^2, proportion of variance explained; F, F-statistic; P, permutation-based P-value. Significant P-values ($p < 0.05$) were highlighted in bold.

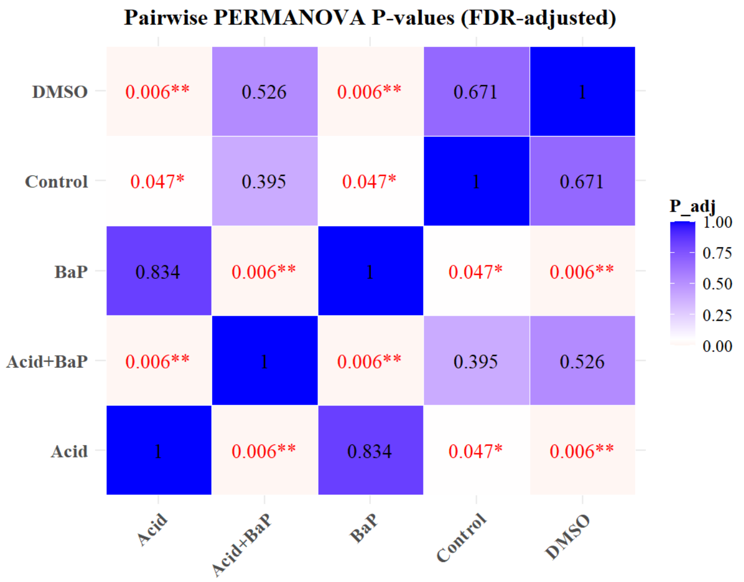

Figure S5. Matrix of pairwise PERMANOVA P-values for treatment groups of coral-associated bacteria.

Table S2. Comparative changes in the relative abundance of abundant dominant core bacterial taxa relative to the control group.

	Asymptotic significance (two-sided) P_value				
Control	DMSO	Acid	BaP	Acid+BaP	Comparison between groups
Sphingomonadaceae	1.000	0.072	**0.012**	1.000	**0.001**
Burkholderiaceae	1.000	0.152	0.105	1.000	**0.002**
Unclassified taxa	-	-	-	-	0.623
Lactobacillaceae	-	-	-	-	0.559
Rhodocyclaceae	0.976	0.782	0.200	**0.033**	**0.001**
Beijerinckiaceae	1.000	1.000	0.338	1.000	**0.001**
Moraxellaceae	-	-	-	-	0.369
Comamonadaceae	-	-	-	-	0.651
Pseudomonadaceae	1.000	**0.013**	**0.002**	1.000	**0.000**
Rhizobiaceae	1.000	0.882	1.000	1.000	**0.044**
Enterobacteriaceae	-	-	-	-	0.053
Chitinophagaceae	0.308	1.000	0.525	0.895	**0.028**
Streptococcaceae	1.000	**0.001**	**0.014**	1.000	**0.005**

Note: The significance values were adjusted for multiple comparisons using the Bonferroni correction.

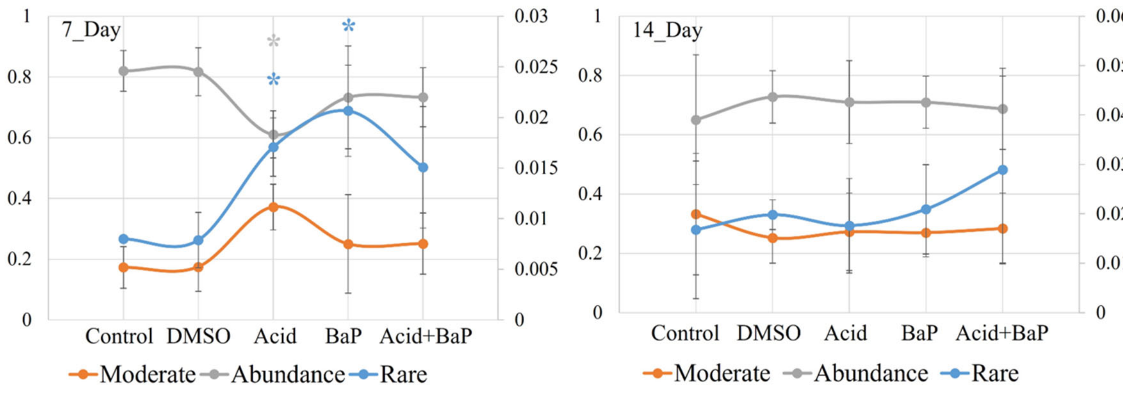

Figure S6. Differences in the relative abundance of abundant, rare and moderate of coral-associated bacteria in different treatment groups. Abundance, represented abundance of bacterial community whose relative abundance ≥ 1 %; Rare, represented rare bacterial communitywhose relative abundance ≤ 0.01 %; Moderate, represented moderate bacterial community with a relative abundance between rare and abundant. Error bars represent standard deviation (SD); "*" represented a significant difference ($p < 0.05$) compared with the control groups. P values were adjusted using the Bonferroni correction for multiple comparisons.

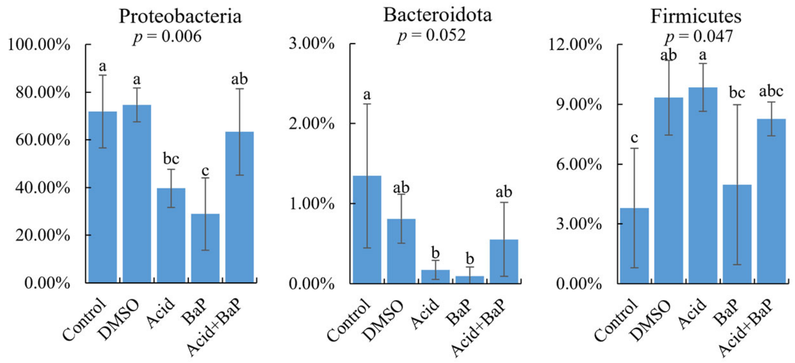

Figure S7. Comparison of OTUs relative abundance ratio at phylum level in different treatment groups. Control, Control group; DMSO, Solvent control group; Acid, Acidification treatment group; BaP, BaP treatment group; Acid+BaP, Combined treatment group.
